# Structure and In Vitro Bioactivity against Cancer Cells of the Capsular Polysaccharide from the Marine Bacterium *Psychrobacter marincola*

**DOI:** 10.3390/md18050268

**Published:** 2020-05-19

**Authors:** Maxim S. Kokoulin, Alexandra S. Kuzmich, Lyudmila A. Romanenko, Irina V. Chikalovets, Oleg V. Chernikov

**Affiliations:** 1 G.B. Elyakov Pacific Institute of Bioorganic Chemistry, Far Eastern Branch, Russian Academy of Sciences, 159/2, Prospect 100 let Vladivostoku, Vladivostok 690022, Russia; assavina@mail.ru (A.S.K.); lro@piboc.dvo.ru (L.A.R.); ivchik6@mail.ru (I.V.C.); chernikov@piboc.dvo.ru (O.V.C.); 2 Far Eastern Federal University, 8, Sukhanova str., Vladivostok 690950, Russia

**Keywords:** marine bacteria, *Psychrobacter*, capsular polysaccharide, pseudaminic acid, lactic acid, antiproliferative activity, HL-60

## Abstract

*Psychrobacter marincola* KMM 277^T^ is a psychrophilic Gram-negative bacterium that has been isolated from the internal tissues of an ascidian *Polysyncraton* sp. Here, we report the structure of the capsular polysaccharide from *P. marincola* KMM 277^T^ and its effect on the viability and colony formation of human acute promyelocytic leukemia HL-60 cells. The polymer was purified by several separation methods, including ultracentrifugation and chromatographic procedures, and the structure was elucidated by means of chemical analysis, 1-D, and 2-D NMR spectroscopy techniques. It was found that the polysaccharide consists of branched hexasaccharide repeating units containing two 2-*N*-acetyl-2-deoxy-d-galacturonic acids, and one of each of 2-*N*-acetyl-2-deoxy-d-glucose, d-glucose, d-ribose, and 7-*N*-acetylamino-3,5,7,9-tetradeoxy-5-*N*-[(*R*)-2-hydroxypropanoylamino]- l-*glycero*-l-*manno*-non-2-ulosonic acid. To our knowledge, this is the first finding a pseudaminic acid decorated with lactic acid residue in polysaccharides. The biological analysis showed that the capsular polysaccharide significantly reduced the viability and colony formation of HL-60 cells. Taken together, our data indicate that the capsular polysaccharide from *P. marincola* KMM 277^T^ is a promising substance for the study of its antitumor properties and the mechanism of action in the future.

## 1. Introduction

Many marine bacteria can produce extracellular polysaccharides (EPSs). Bacterial EPSs usually occur in two forms: As capsular polysaccharides (CPSs), if they are associated with the cell surface, and medium-released polysaccharides (MRPs), if they are completely released in the environment [1]. The presence of these biopolymers indicates their specific properties and functions that are beneficial to microorganisms; they play an essential role in protecting the bacterial cell from harsh environmental conditions (salinity, temperature, and the availability of nutrients), in surface adhesion (usually through the biofilms formation), intercellular signal transduction, and in resisting the host’s immune response. All these features help to survive and protect their producers from the complex marine environment [1,2].

Bacterial EPSs are bioglycans, mainly consisting of different monosaccharides or its derivatives. In addition, various types of organic and inorganic substituents (amino acids, acetates, pyruvates, lactates, phosphates, and sulfates) can decorate the main linear or branched polysaccharide chain [1,2]. Such a high degree of variability implies the existence of a huge number of bacterial polysaccharides, in which structural changes are almost unlimited.

In recent decades, the growing demand for natural carbohydrate polymers for industrial use has led to significant interest in polysaccharides produced by microorganisms. Additionally, EPSs from marine bacteria have prominent biological activity, including immunomodulatory, antiviral, as well as antitumor activities [3,4,5].

The genus *Psychrobacter* belongs to the class Gammaproteobacteria and includes both psychrophilic and psychrotolerant, halotolerant, and Gram-negative bacteria, which are associated primarily with the Antarctic and marine habitats [6]. Members of *Psychrobacter* have been isolated from various sources, including the Antarctic sea ice, permafrost, marine sediments, deep and surface seawater, fish, and marine invertebrates. Several species have also been isolated from food, clinical sources, poultry, and livestock [7]. At the time of writing, the genus *Psychrobacter* comprises 41 validly described species [8].

The structure of polysaccharides associated with the cell wall of bacteria of the genus *Psychrobacter* has not been studied intensively. To date, only two O-specific polysaccharide structures from *P. muricolla* 2pS^T^ [9] and *P. cryohalolentis* K5^T^ [10], and two capsular polysaccharide structures from *P. arcticus* 273-4 [11] and *P. maritimus* 3pS [12] have been reported.

In this paper, we report on the isolation, purification, and structure of the CPS produced by *P. marincola* KMM 277^T^, isolated from the homogenized internal tissues of an ascidian *Polysyncraton* sp. [13]. It was found that the CPS is a branched high-molecular-weight polymer that effectively inhibits the viability and colony formation of HL-60 cells.

## 2. Results

### 2.1. Isolation, Purification, and General Characterization of the CPS

**Figure 1:**
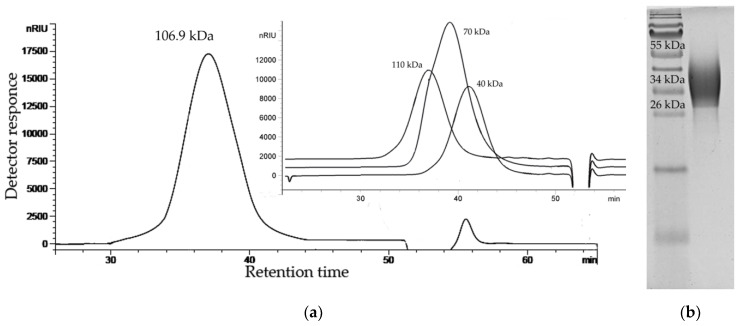
(**a**) HPSEC elution profile of the CPS from *P. marincola* KMM 277^T^ and dextran standards (insert); (**b**) Alcian blue-stained electrophoregram of the CPS from *P. marincola* KMM 277^T^.

An experiment using transmission electron microscopy showed the presence of a capsular structure around *P. marincola* KMM 277^T^ cells [13]. The dried bacterial cells were subjected to extraction with sodium chloride solution followed by enzymatic treatment. The CPS from the resulting material was purified by ultracentrifugation followed by anion-exchange chromatography and gel filtration. Analysis of the CPS revealed no contaminant proteins and fatty acids. The size exclusion chromatography (SEC) analysis showed that the CPS forms a single symmetrical peak and has a molecular weight of about 106.9 kDa (Figure 1a). The electrophoretic profile of the CPS showed a broad smear, limited from 55 (top) to 26 kDa (Figure 1b).

**Figure 2:**
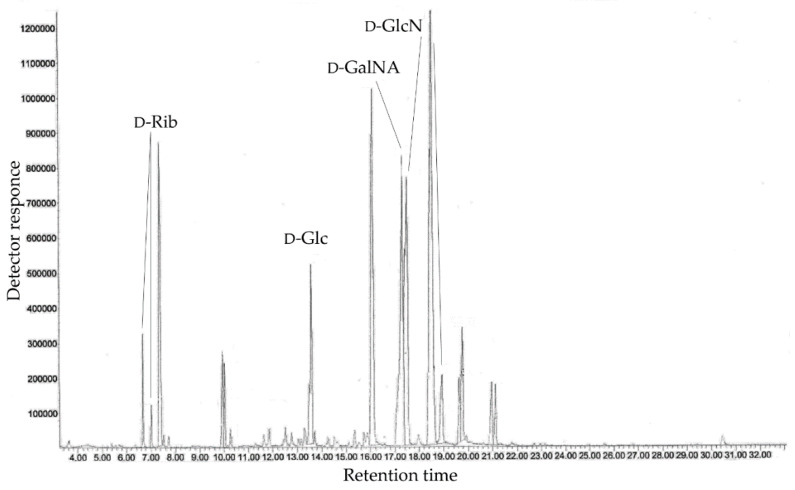
GC profile of the acetylated methyl glycosides derived from the CPS.

Monosaccharide analysis using GC-MS of the acetylated alditols and acetylated methyl glycosides (Figure 2) disclosed the presence of ribose, glucose, glucosamine, and galactosamine uronic acid, all with the D configuration [14,15]. Further study of the CPS by NMR spectroscopy revealed the presence of one more component, 7-*N*-acetylamino-3,5,7,9-tetradeoxy-5-*N*-[(*R*)-2-hydroxypropanoylamino]- l-*glycero*-l-*manno*-non-2-ulosonic acid (see below). The *R* configuration of lactic acid (*R*-Lac) was determined by GC of the acetylated (*S*)-2-butyl ester as described [16]. Methylation analysis of the CPS resulted in the identification of 3,5-di-*O*-methyl-d-ribose (derived from 2-substituted d-ribofuranose residue), 3,4,6-tri-*O*-methyl-d-glucose (derived from 2-substituted d-glucopyranose residue), and 2-deoxy-4,6-di-O-methyl-2-(*N*-methylacetamido)-d-glucose (derived from 3-substituted d-glucosamine residue).

**Figure 3:**
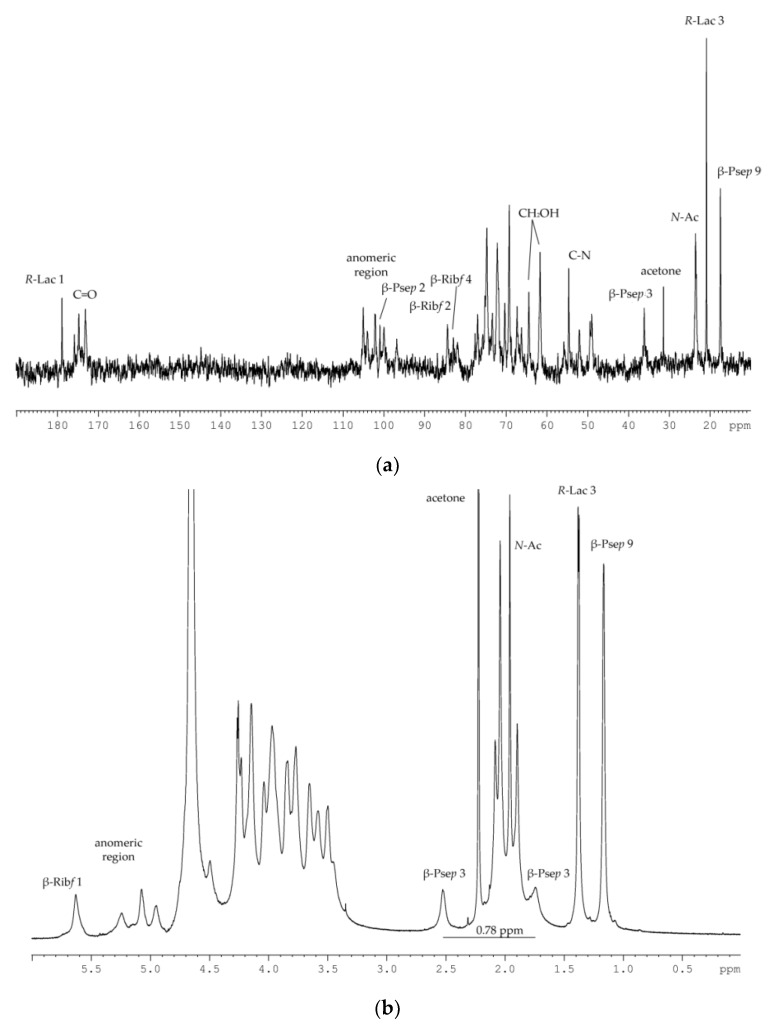
(**a**) ^13^C NMR spectrum of the CPS from *P. marincola* KMM 277^T^; (**b**) ^1^H NMR spectrum of the CPS from *P. marincola* KMM 277^T^.

The ^13^C NMR spectrum of the CPS (Figure 3a) displayed, inter alia, signals of six anomeric carbons at *δ_C_* 105.1, 104.1, 102.2, 101.0 (quaternary carbon, data of the DEPT-135 experiment), 100.0, and 96.8; five nitrogen-bearing carbons at *δ_C_* 55.9, 54.7, 52.1, 49.5, and 49.1; three hydroxymethyl groups (data of the DEPT-135 experiment) at *δ_C_* 64.5 and 61.7 (double intensity); methylene carbon (data of the DEPT-135 experiment) at *δ_C_* 36.2; two methyl groups at *δ_C_* 20.9 and 17.5; four methyl groups of *N*-acetyl substituents at *δ_C_* 23.4–23.8; and numerous carbonyl carbons at *δ_C_* 173.1–178.9. These data demonstrated a hexasaccharide repeating unit of the CPS and suggested the presence of an amide-linked *R*-Lac residue (characteristic signals at *δ_C_* 178.9, 69.2, and 20.9).

Accordingly, the ^1^H NMR spectrum of the CPS (Figure 3b) revealed, inter alia, signals of five protons in the anomeric region at *δ_H_* 5.63–4.59, one methylene group at *δ_H_* 2.53 and 1.74, four *N*-acetyl groups at *δ_H_* 2.08–1.90, and two methyl groups at *δ_H_* 1.38 (H-3 of *R*-Lac residue, ^3^*J*_2,3_ 6.2 Hz) and 1.16. The last signal together with signals of the methylene group and data of the ^13^C NMR spectrum indicated the presence of 5,7-amino-3,5,7,9-tetradeoxy-L-*glycero*-L-*manno*-non-2-ulosonic acid (pseudaminic acid, Pse*p*) residue (the full identification was achieved on the basis of *^3^J_H,H_* coupling constant values and ^1^H and ^13^C NMR chemical shifts obtained for the low molecular weight derivative, see below). A relatively large difference (0.78 ppm) between the chemical shifts of H-3_ax_ and H-3_eq_ was typical for the axial orientation of the carboxyl group of 3-deoxynon-2-ulosonic acids [17], and therefore the Pse*p* residue was β-linked. In addition, the presence of the signal at *δ_H_* 5.63 together with the signals at *δ_C_* 83.0 and 84.4 in the ^13^C NMR spectrum of the CPS corresponded to the 2-substituted β-d-ribofuranose residue (β-d-Rib*f*) and was in accordance with the data of the methylation analysis [18]. 

Unfortunately, the poor quality of the two-dimensional (2-D) NMR spectra (signal densities were broad and mostly lost in the baseline noise) prevented any conclusive structural assignment by the application of 2-D techniques. In this connection, the CPS was subjected to a mild acid degradation and three components, one high molecular weight polysaccharide (HMP) and two oligosaccharides (OS-1 and OS-2), were isolated by gel filtration. 

### 2.2. Structural Elucidation of the CPS

**Figure 4:**
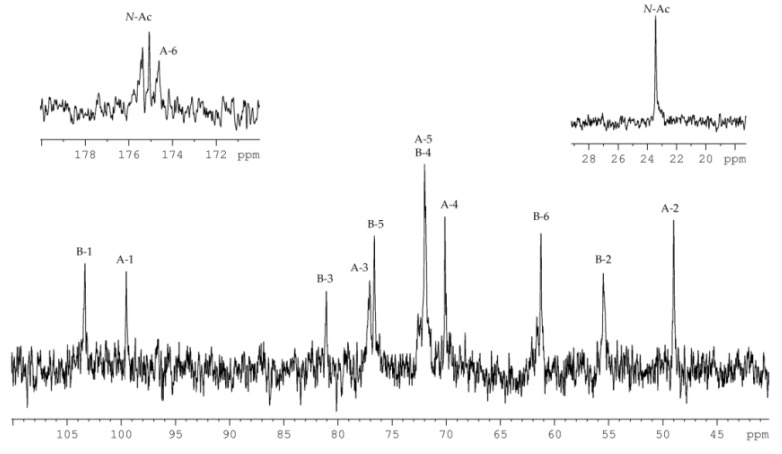
^13^C NMR spectrum of the HMP. Numerals refer to carbons in sugar residues denoted by capital letters as described in Table 1. *N*-Ac stands for the *N*-acetyl group.

The ^13^C NMR spectrum of the HMP (Figure 4) contained signals of two anomeric carbons at *δ_C_* 103.3 and 99.5, thus indicating a disaccharide repeating unit; two nitrogen-bearing carbons at *δ_C_* 55.5 and 49.0; one hydroxymethyl group (data of the DEPT-135 experiment) at *δ_C_* 61.3; one carboxyl carbon at *δ_C_* 174.6; carbons of two *N*-acetyl groups at *δ_C_* 23.4 (CH_3,_ double intensity), 175.1 and 175.4 (CO); and other carbons at *δ_C_* 70.1–81.1.

The ^1^H NMR spectrum of the HMP revealed the characteristic signals of two protons in the anomeric region at *δ_H_* 5.38 and 4.60, two *N*-acetyl groups at *δ_H_* 2.04 and 1.94, and other sugar ring protons in the region of *δ_H_* 3.42–4.50.

The ^1^H and ^13^C NMR spectra of the HMP were assigned by ^1^H,^1^H-COSY, ^1^H,^1^H-TOCSY, ^1^H,^1^H-ROESY, ^1^H,^13^C-HSQC, and ^1^H,^13^C-HMBC experiments, and the chemical shifts are presented in Table 1. 

The ^1^H,^1^H-COSY, and ^1^H,^1^H-TOCSY spectra revealed proton spin systems for one sugar residue having the *galacto* configuration (H-1 up to H-4, and H-4/H-5 correlations) and one having the *gluco* configuration (correlations between all ring proton). The sugar residue with the *galacto* configuration was identified as d-Gal*p*NAcA (**A**) based on H-2/C-2 correlation at *δ_H_*/*δ_C_* 4.29/49.0 in the ^1^H,^13^C-HSQC spectrum, and H-5/C-6 correlation at *δ_H_*/*δ_C_* 4.23/174.6 in the ^1^H,^13^C-HMBC spectrum. The sugar residues with the *gluco* configuration were identified as d-Glc*p*NAc (**B**) based on H-2/C-2 and H-6/C-6 correlations at *δ_H_*/*δ_C_* 3.78/55.5 and *δ_H_*/*δ_C_* 3.91, 3.78/61.3, respectively, in the ^1^H,^13^C-HSQC spectrum. The ^1^*J*_C1–H1_ coupling constant values determined from the gated-decoupling spectrum of the HMP showed that d-Gal*p*NAcA was α-linked (>170 Hz), whereas d-Gal*p*NAcA was β-linked (<165 Hz). 

**Figure 5:**
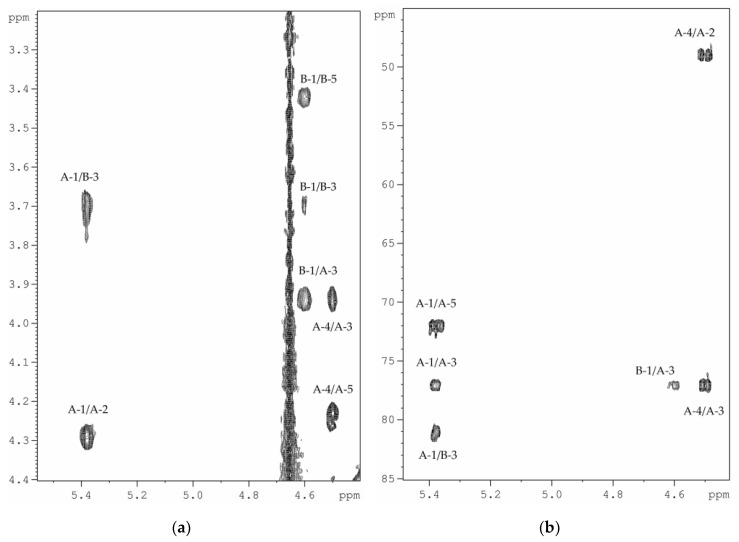
(**a**) Part of ^1^H, ^1^H-ROESY spectrum of the HMP; (**b**) Part of ^1^H,^13^C-HMBC spectrum of HMP. Numerals refer to protons and carbons in sugar residues denoted by capital letters as described in Table 1.

Linkage analysis of the HMP was performed using the ^1^H,^1^H-ROESY (Figure 5a), and ^1^H,^13^C-HMBC experiments (Figure 5b). The ^1^H,^1^H-ROESY spectrum revealed the following cross-peaks: H-1 α-d-Gal*p*NAcA (**A**)/H-3 β-d-Glc*p*NAc (**B**) at *δ_Н_*/*δ_Н_* 5.38/3.71, and H-1 β-d-Glc*p*NAc (**B**)/H-3 α-d-Gal*p*NAcA (**A**) at *δ_Н_*/*δ_Н_* 4.60/3.95. Accordingly, the ^1^H,^13^C-HMBC spectrum displayed the following cross-peaks: H-1 α-d-Gal*p*NAcA (**A**)/C-3 β-d-Glc*p*NAc (**B**) at *δ_Н_*/*δ_C_* 5.38/81.1, and H-1 β-d-Glc*p*NAc (**B**)/C-3 α-d-Gal*p*NAcA (**A**) at *δ_Н_*/*δ_C_* 4.60/77.1. These data showed that both monosaccharide residues were 3-substituted.

Based on the data obtained, it was concluded that HMP consists of disaccharide repeating units with the following structure:

→3)-α-d-GalpNAcA-(1→3)-β-d-GlcpNAc-(1→

**Figure 6:**
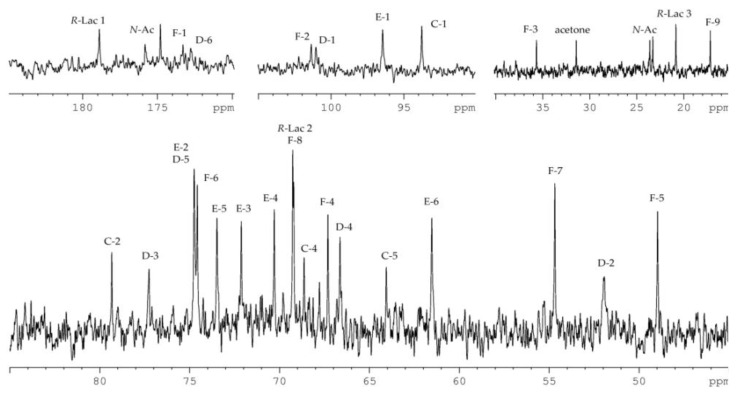
^13^C NMR spectrum of the OS-1. Numerals refer to carbons in sugar residues denoted by capital letters as described in Table 2. *N*-Ac stands for the *N*-acetyl group.

In turn, the ^13^C NMR spectrum of the OS-1 (Figure 6) contained signals of four anomeric carbons at *δ_C_* 101.4, 101.0, 96.4, and 93.8; three nitrogen-bearing carbons at *δ_C_* 54.7, 51.9, and 49.0; two hydroxymethyl groups (data of the DEPT-135 experiment) at *δ_C_* 64.1 and 61.5; methylene carbon (data of the DEPT-135 experiment) at *δ_C_* 35.7; two methyl groups at *δ_C_* 20.9 and 17.2; three carboxyl carbons at *δ_C_* 178.9, 173.4, and 172.8; carbons of two *N*-acetyl groups at *δ_C_* 23.6, 23.3 (CH_3_) and 175.9, 174.9 (CO); and other carbons at *δ_C_* 66.6–79.3. 

The ^1^H NMR spectrum of the OS-1 revealed the characteristic signals of three protons in the anomeric region at *δ_Н_* 5.13 (*^3^J_1,2_* 5.7 Hz), 5.06 (*^3^J_1,2_* 3.7 Hz), and 4.80 (*^3^J_1,2_* 8.3 Hz); one methylene group at *δ_Н_* 2.52 (*^3^J_3eq,4_* 4.8 Hz) and 1.76 (*^3^J_3ax,4_* 12.9 Hz); two methyl groups at *δ_Н_* 1.38 (*^3^J_2,3_* 7.0 Hz) and 1.17 (*^3^J_8,9_* 6.5 Hz); two *N*-acetyl groups at *δ_Н_* 2.05 and 1.95; and other protons at *δ_Н_* 3.51–4.55. 

The ^1^H and ^13^C NMR spectra of the OS-1 were assigned by 2D NMR experiments, and the chemical shifts are presented in Table 2.

Based on the *^3^J_H,H_* coupling constant values and ^1^H and ^13^C NMR chemical shifts, spin systems for one residue of each of β-d-Rib*p* (**C**, major form at the reducing end), β-d-Gal*p*NAcA (**D**), α-d-Glc*p* (**E**), β-Pse*p* (**F**), and *R*-Lac were identified. In detail, the β-d-Gal*p*NAcA was confirmed based on H-2/C-2 correlation at *δ_H_*/*δ_C_* 4.05/51.9 in the ^1^H,^13^C-HSQC spectrum, and H-5/C-6 correlation at *δ_H_*/*δ_C_* 4.39/172.8 in the ^1^H,^13^C-HMBC spectrum. The β-Pse*p* was confirmed by H-3_ax_/C-1, H-3_ax_/C-2, and H-3_eq_/C-2 correlations at *δ_Н_*/*δ_C_* 1.76/173.4, 1.76/101.4, and 2.52/101.4, respectively, in the ^1^H,^13^C-HMBC spectrum (Figure 6). The ^1^H,^13^C-HSQC spectrum showed cross-peaks of H-5 and H-7 of β-Pse*p* with nitrogen-bearing carbons at *δ_H_*/*δ_C_* 4.22/49.0 and 4.06/54.7, respectively. The ^1^H and ^13^C NMR chemical shifts of the β-Pse*p* and *^3^J_H,H_* coupling constants (*^3^J_3ax,4_* 12.9 Hz, *^3^J_4,5_* 4.2 Hz, *^3^J_5,6_* 2.1 Hz, *^3^J_6,7_* 10.3 Hz) were consistent with a l-*glycero*-l-*manno* configuration and differed from the spectral parameters of the other known stereoisomers [17].

**Figure 7:**
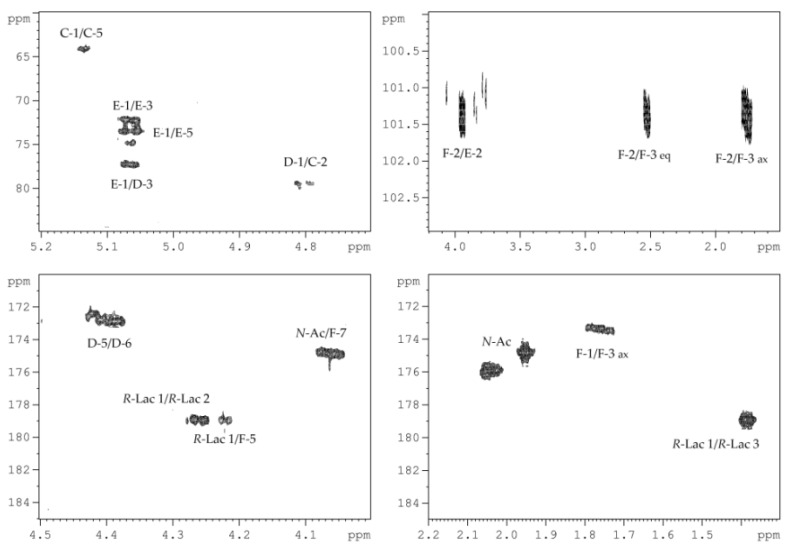
Fragments of ^1^H,^13^C-HMBC spectrum of the OS-1. Numerals refer to carbons in sugar residues denoted by capital letters as described in Table 2. *N*-Ac stands for the *N*-acetyl group.

The position of substitution and the sequence of the sugar residues in the OS-1 were established using the ^1^H,^13^C-HMBC experiment (Figure 7). The ^1^H,^13^C-HMBC spectrum displayed the following inter-residue cross-peaks: C-2 β-Pse*p*/H-2 α-d-Glc*p* at *δ_C_/δ_H_* 101.4/3.94, H-1 α-d-Glc*p/*C-3 β-d-Gal*p*NAcA at *δ_Н_/δ_C_* 5.06/77.3, and H-1 β-d-Gal*p*NAcA/C-2 β-d-Rib*p* at *δ_Н_/δ_C_* 4.80/79.3. These data indicated that β-d-Rib*p* and α-d-Glc*p* were 2-substituted, β-d-Gal*p*NAcA was 3-substituted, and β-Pse*p* had no substitution and was at the non-reducing end of the OS-1. In addition, the correlation between H-5 of β-Pse*p* and C-1 of *R*-Lac at *δ_Н_/δ_C_* 4.22/178.9 in the ^1^H,^13^C-HMBC spectrum indicated the location of *R*-Lac at position 5 of β-Pse*p*. 

Thus, it was concluded that OS-1 was a tetrasaccharide with the following structure:

β-Pse*p*5(*R*-Lac)7Ac-(2→2)-α-d-Glc*p*-(1→3)-β-d-Gal*p*NAcA-(1→2)-β-d-Rib*p*

Studies using 1-D and 2-D NMR spectroscopy (Table 2) showed that OS-2 differed from the OS-1 in the absence of acid-labile β-Pse*p*5(*R*-Lac)7Ac residue and had the following structure:

α-d-Glc*p*-(1→3)-β-d-Gal*p*NAcA-(1→2)-β-d-Rib*p*

All the data obtained indicated that the backbone of CPS consisted of disaccharide repeating units (according to the structure of the HMP) to which the tetrasaccharide side branch (according to the structure of the OS-1) was attached via a glycosidic bond formed by the β-d-Rib*f* residue (according to the ^1^H, ^13^C NMR and methylation analysis data for the CPS).

Attempts to obtain a product with improved spectroscopic properties by selective cleavage of β-Pse*p*5(*R*-Lac)7Ac residue during hydrolysis of the CPS with acetic acid led to the simultaneous cleavage of the glycosidic bond formed by the β-d-Rib*f* residue. The β-Pse*p*5(*R*-Lac)7Ac and sidechain were only partially removed, creating complex heterogeneity in the polymer and making it difficult to study.

In order to determine the location of the sidechain, the CPS was subjected to Smith degradation, and the modified CPS (MPS) was isolated by gel-filtration. As expected, 2-substituted α-d-Glc*p* residue was oxidized and its glycosidic linkage was then cleaved selectively under mild acid conditions, whereas the other sugar residues and their linkages mostly remained unaffected. The structure of the MPS was established using 2-D NMR spectroscopy, as described above for the HMP and OS-1 (Table 3). 

**Figure 8:**
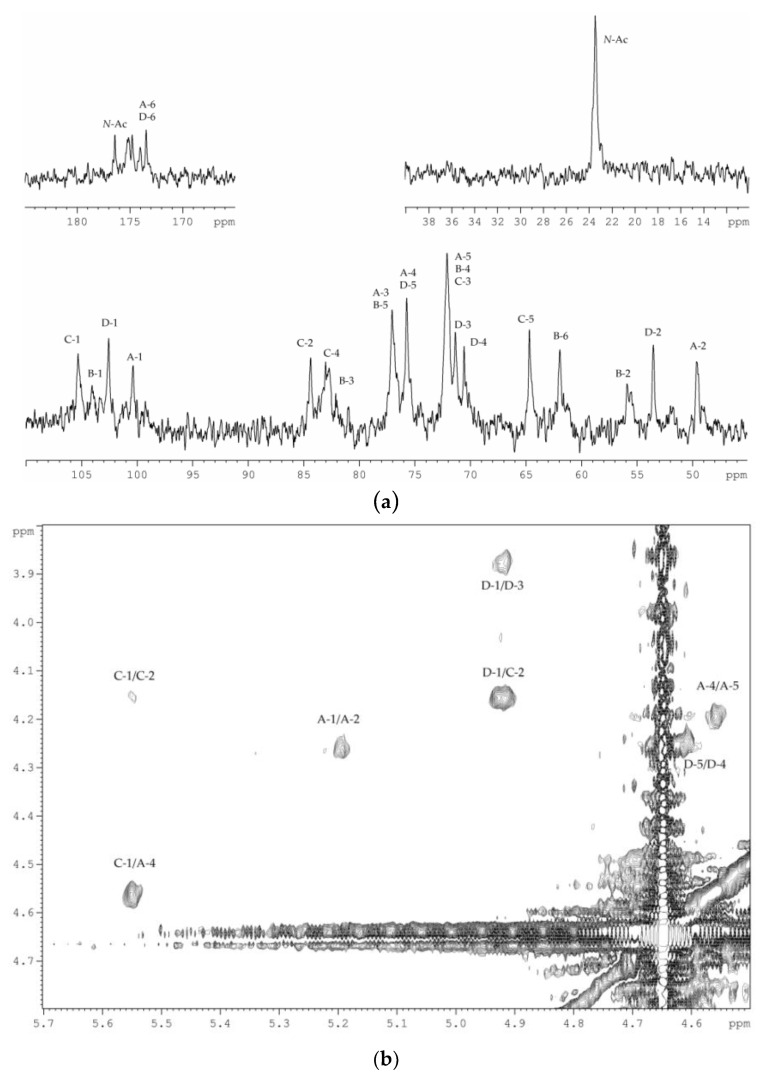
(**a**) ^13^C NMR spectrum of the MPS; (**b**) Part of ^1^H, ^1^H-ROESY spectrum of the MPS. Numerals refer to protons and carbons in sugar residues denoted by capital letters as described in Table 3. *N*-Ac stands for the *N*-acetyl group.

The ^13^C NMR spectrum of the MPS (Figure 8a) contained signals of four anomeric carbons at *δ_C_* 105.3, 104.1, 102.6, and 100.4; three nitrogen-bearing carbons at *δ_C_* 55.9, 53.5, and 49.6; two hydroxymethyl groups (data of the DEPT-135 experiment) at *δ_C_* 64.7 and 61.9; two carboxyl carbons at *δ_C_* 174.0 and 173.5; carbons of *N*-acetyl groups at *δ_C_* 23.5 (CH_3_) and 176.4–174.8 (CO); and other carbons at *δ_C_* 70.6–84.4. The signals of β-Pse*p*5(*R*-Lac)7Ac and α-d-Glc*p* were absent. Significant downfield displacement of the signal for C-4 of α-d-Gal*p*NAcA at *δ_C_* 75.7, as compared with its position in the ^13^C NMR spectrum of the HMP, was due to glycosylation of this sugar residue at *O*-4. This finding was confirmed by the presence of a correlation between H-1 of β-d-Rib*f* and H-4 of α-d-Gal*p*NAcA at *δ_Н_*/*δ_Н_* 5.55/4.56 in the ^1^H,^1^H-ROESY spectrum (Figure 8b).

Taken together, these data led to the determination of the structure of the branched high-molecular-weight CPS from the marine bacterium *P. marincola* KMM 277^T^:
→3)-β-d-Glc*p*NAc-(1→3)-α-d-Gal*p*NAcA-(1→4










↑










1










β-Pse*p*5(*R*-Lac)7Ac-(2→2)-α-d-Glc*p*-(1→3)-β-d-Gal*p*NAcA-(1→2)-β-d-Rib*f*













### 2.3. In Vitro Effect of the CPS on Cell Viability, and Colony Formation of Cancer Cells

MTS assay is often used to determine whether test molecules in vitro affect proliferation and cell viability. In the present study, the determination of the cell viability effect of the CPS from the marine bacterium *P. marincola* KMM 277^T^ was performed on human colorectal adenocarcinoma HT-29, Burkitt’s lymphoma Raji, acute monocytic leukemia THP-1, and acute promyelocytic leukemia HL-60 cells.

**Figure 9:**
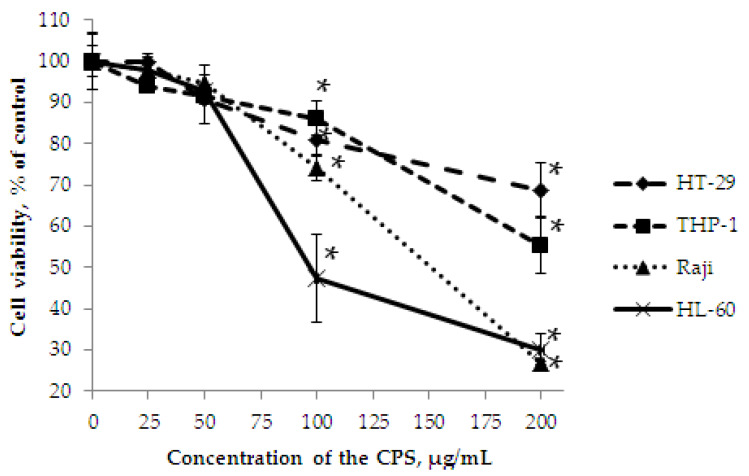
The viability of HT-29, THP-1, Raji, and HL-60 cells treated with the CPS using the MTS assay. Data are represented as the means ± SD as determined from triplicate experiments. *p < 0.05.

It was shown that the CPS had weak cytotoxic activity (Figure 9) against HT-29 and THP-1 cells, the inhibition was less than 20% at concentrations of up to 100 μg/mL after 72 h of treatment. At a concentration of 200 μg/mL, the CPS showed inhibition of cells by 31% and 45%, respectively. However, the experiment under the same conditions indicated that the CPS significantly inhibited the viability of Raji and HL-60 cells. At a concentration of 200 μg/mL, the CPS showed inhibition of 73% and 70%, respectively; moreover, in the case of HL-60 cells, the half-maximal inhibitory concentration (IC_50_) was 97.3 μg/mL.

**Figure 10:**
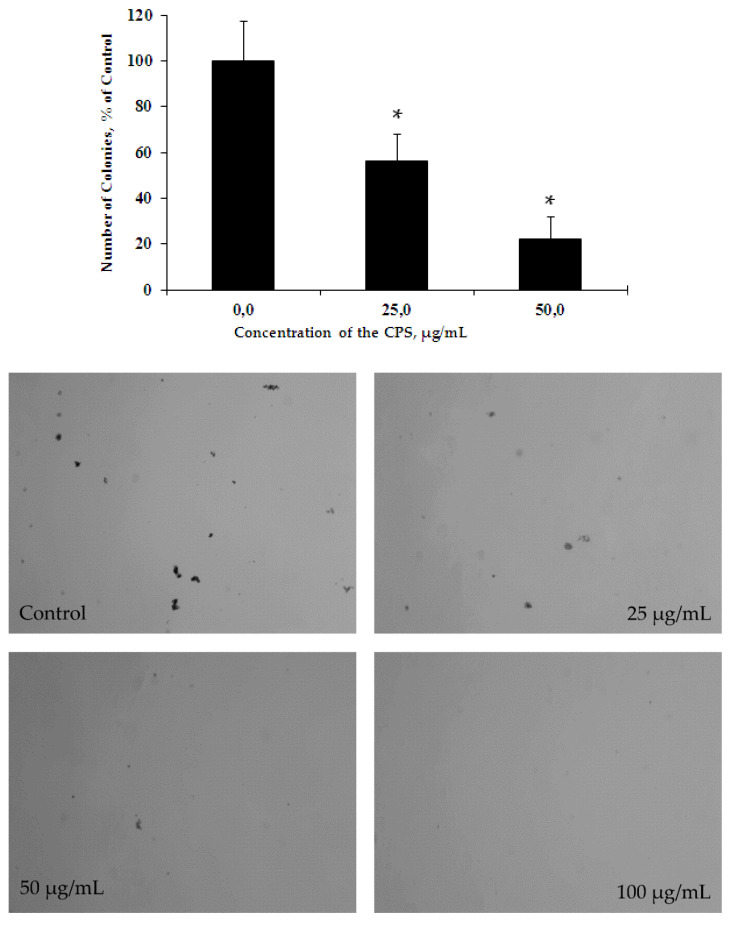
The inhibitory effect of the CPS on colony formation of HL-60 cells versus untreated control cells. Data are represented as the means ± SD as determined from triplicate experiments. *p < 0.05.

The soft agar colony formation assay, which measures the ability of cells to proliferate in semi-solid matrices, provides a tool to test the effects of novel compounds on cell proliferation and migration. In the next step of our study, we determined the inhibition of colony formation of the HL-60 cells using the soft agar assay (Figure 10). HL-60 cells were treated with the CPS at concentrations of 25–200 μg/mL and incubated for 10 days. It was shown, that the CPS also effectively inhibited the colony formation of the studied cells in a dose-dependent manner with the inhibition concentration (INCC_50_) of 31.0 μg/mL. It is important to note that in this test, the CPS acted at concentrations three times less than in the MTS assay. At concentrations of 100–200 μg/mL, no colonies were detected. 

Thus, we demonstrated that the CPS from *P. marincola* KMM 277^T^ was influenced both on cell viability and colony formation of human acute promyelocytic leukemia HL-60 cells in a dose-dependent manner.

## 3. Discussion

In this study, we determined the structure of the CPS isolated from the marine Gram-negative bacterium *P. marincola* KMM 277^T^ and showed that it is presented as a branched high molecular weight polymer. As far as we know, this structure has not been previously detected in bacterial polysaccharides [19]. The hexasaccharide repeating unit of the CPS from *P. marincola* KMM 277^T^ contains two 2-*N*-acetyl-2-deoxy-d-galacturonic acids, 2-*N*-acetyl-2-deoxy-d-glucose, d-glucose, d-ribose, and a novel constituent component of bacterial glycopolymers – 7-*N*-acetylamino-3,5,7,9-tetradeoxy-5-*N*-[(*R*)-2-hydroxypropanoylamino]-l-*glycero*-l-*manno*-non-2-ulosonic acid.

Previously, various pseudaminic acid derivatives have been found in numerous carbohydrate-containing biopolymers associated with bacterial cell walls [17,20]. Among marine Gram-negative bacteria, pseudaminic acid derivatives were found in polysaccharides from *Pseudoalteromonas distincta* KMM 638 [21], *P. atlantica* IAM 14165 [22], and *Cellulophaga fucicola* NN015860^T^ [23]. It is important to note that another member of the *Psychrobacter* genus, *P. arcticus* 273-4, produces a CPS containing 5,7-di-*N*-acetyl-pseudaminic acid [11]. In both cases, pseudaminic acid derivatives occupy a lateral position, and perhaps this is important for bacterial survival and niche adaptation [11]. In addition, a polysaccharide with a similar architecture consisting of a disaccharide backbone and a rather long tetrasaccharide sidechain has been described for *P. cryohalolentis* K5^T^ [10].

Cancer remains one of the leading causes of death worldwide and represents a diverse group of diseases characterized by the uncontrolled proliferation of anaplastic cells. The search for compounds with antitumor activity is of great interest in connection with an increase in the number and high mortality in cancer patients. The anticancer effect has previously been demonstrated for several polysaccharides obtained from marine and extremophilic bacteria [1,24,25]. A study of the biological activity of the CPS from *P. marincola* KMM 277^T^ showed that it has a selective effect on the cell viability and colony formation of HL-60 cells.

The inhibitory effect on HL-60 cells has also been shown for other polysaccharides of marine origin. For example, the sulfated polysaccharide from a brown alga *Ecklonia cava* suppressed the proliferation of cancer cells with an IC_50_ of 100.0 μg/mL [26]. The fucoidan fraction purified from *Sargassum polycystum* induced apoptosis via the mitochondria-mediated pathway in HL-60. It is interesting to note that in the cell viability test, fucoidan showed an antiproliferative effect with an IC_50_ of 84.6 μg/mL [27]. Apoptosis induction of HL-60 cells has also been demonstrated for polysaccharides from *Fucus vesiculosus* [28], *Sargassum coreanum* [29], and *Hydroclathrus clathratus* [30]. Among marine microorganisms, inhibitory activity against HL-60 cells has been shown for exopolysaccharide from endogenous fungus *Alternaria* sp. SP-32. The polysaccharide consisting of a mannan core and a galactoglucan chain exhibited cytotoxicity to HL-60 cells with an IC_50_ of 143.0 μg/mL [31]. Regarding the inhibition of clonogenicity of the HL-60 cell line using the soft agar assay, a polysaccharide from *Ganoderma lucidum* was studied. The polysaccharide, which is β-d-glucan, did not have a carcinogenic effect even at a high dose of 400 μg/mL [32].

These data indicate that the CPS from *P. marincola* KMM 277^T^ is comparable in its effect to such well-known molecules as fucoidans and is a promising substance for further studies of its antitumor properties and mechanism of action.

## 4. Materials and Methods 

### 4.1. Isolation and Purification of the CPS

*P. marincola* KMM 277^T^ was obtained from the Collection of Marine Microorganisms (KMM) of the G.B. Elyakov Pacific Institute of Bioorganic Chemistry, Far Eastern Branch of Russian Academy of Sciences (Vladivostok, Russia). The bacteria were cultivated as described [33]. Dry bacterial cells (5.4 g) were suspended in 150 mL of 0.15 M NaCl and heated at 80 °C with stirring for 30 min. The cell pellet was collected by centrifugation (5000 rpm, 25 min, 4 °C) and the isolation procedure was repeated two more times. Supernatants were combined, dialyzed (MWCO 12,000 Da), and lyophilized to give crude CPS (935 mg). The freeze-dried material (340 mg) was resuspended in 30 mL of digestion buffer containing TRIS/EDTA (0.01 M/0.001 M) and 0.01 M MgCl_2_. After the addition of 4 mg of RNAse and DNAse (Sigma), the solution was incubated for 16 h at 37 °C, followed by 60 °C for 2 h after the addition of 2 mg of proteinase K (Sigma). After dialysis against distilled water (MWCO 12000 Da), it was lyophilized to give enzymatic-treated CPS (193 mg). The material was resuspended in 6 mL of water and centrifuged at 105,000× g for 4 h. The freeze-dried supernatant (116 mg) was purified by anion-exchange chromatography on a column (10 × 1.5 cm) of Toyopearl DEAE-650M in a stepwise gradient of NaCl (0.125, 0.25, 0.5, 1, and 2 M). The resulting main CPS fraction (70 mg, 0.25 M NaCl) was dialyzed and lyophilized. Finally, the polysaccharidic material was loaded on Toyopearl HW-55 column (120 × 1.5 cm) eluted with ammonium hydrogen carbonate (0.05 M), yielding 42 mg of pure CPS. Elution was monitored with a differential refractometer (Knauer, Germany).

### 4.2. Determination of the Molecular Weight and Electrophoretic Analysis of the CPS

Molecular weight of the CPS was analyzed using HPLC (Agilent 1100 Series, Hamburg, Germany), equipped with a successively connected columns of Shodex Asahipak GS-520 HQ and GS-620 HQ (7.5 mm × 300 mm) at 50 °C with elution by 0.15 M NaCl (0.4 mL/min). Columns were calibrated using standard dextrans of 6, 12, 40, 70, and 100 kDa (Sigma, St. Louis, MO, USA). Electrophoresis of the CPS preparation was performed in 15% (w/v) polyacrylamide gel according to Laemmli protocol [34], and bands were visualized by alcian blue. The Protein Plus molecular weight marker (Bio-Rad, Hercules, CA, USA) was used as the standard.

### 4.3. Compositional Analysis of the CPS

Monosaccharide composition was analyzed as the alditol acetates obtained by hydrolysis of the CPS with 2 M CF_3_COOH (120 °C, 2 h) and as the acetylated methyl glycosides obtained by methanolysis of the CPS with 2 M acetylchloride in methanol (120 °C, 4 h) by GC on an Agilent 6850 chromatograph (Santa Clara, CA, USA) equipped with an HP-5 MS capillary column using a temperature program from 150 (3 min) to 230 °C (10 min) at 3 °C min−1. GC-MS was performed on a Hewlett Packard 5890 chromatograph (USA) equipped with a HP-5MS column and connected to a Hewlett Packard 5973 mass spectrometer (USA). The absolute configurations of monosaccharides were determined by GC of the acetylated (*S*)-2-butyl or (*S*)-2-octyl glycosides as described [14,15]. The absolute configuration of lactic acid was determined as described [16]. Methylation analysis of the CPS was performed according to the Hakomori method [35]. Fatty acid analysis was performed by GC of methyl derivatives after methanolysis of the CPS with 2 M acetylchloride in methanol (120° C, 4 h). Proteins were analyzed by the conventional method [36].

### 4.4. Partial Acid Hydrolysis and Smith Degradation of the CPS

The CPS sample (30 mg) was hydrolyzed with 0.1 M HCl (1 mL) for 1 h at 100 °C. After acid removal, the product was fractioned by gel-permeation chromatography on TSK HW-40 in aq 0.1% AcOH to give the HMP (9.2 mg), OS-1 (4 mg), and OS-2 (6 mg). Elution was monitored with a differential refractometer.

The CPS sample (30 mg) was oxidized with 0.1 M NaIO4 (3 mL) in the dark at 20 °C for 72 h. After reduction with an excess of NaBH_4_ and dialysis MWCO 3500 Da), the product was hydrolyzed with 1% CH_3_COOH at 100 °C for 3 h and fractionated by gel-permeation chromatography on TSK HW-50 in aq 0.1% AcOH to give the MPS (9.2 mg). Elution was monitored with a differential refractometer.

### 4.5. NMR Spectroscopy

^1^H and ^13^C NMR spectra of the CPS and its derivatives were recorded on a Bruker Avance-III (700.13 MHz for ^1^H and 176.04 MHz for ^13^C) spectrometer (Germany) at 37 °C using acetone (δ_C_ 31.45, δ_H_ 2.225) as the internal standard. 2-D NMR experiments were performed as described [33].

### 4.6. Biological Activity

#### 4.6.1. Cell Culture

HT-29 (ATCC# HTB-38), THP-1 (ATCC# TIB-202), HL-60 (ATCC# CCL-240), and Raji (ATCC# CCL-86) cells were grown in McCoy’s 5a Medium Modified and RPMI 1640, respectively, supplemented with 10% (v/v) heat-inactivated fetal bovine serum (FBS), 2 mM l-glutamine, and 1% penicillin- streptomycin at 37 °C in a humidified atmosphere containing 5% CO_2_. 

#### 4.6.2. Cell Viability Assay

The cells (6 × 10^3^ cells/well) were cultured for 12 h in 96-well plates in the corresponding medium (100 μL/well) at 37 °C in 5% CO_2_. After that, 100 μL of fresh medium containing the different concentrations (25–200 μg/mL) of the CPS were added, and the cells were incubated for an additional 72 h. Then, 20 μL of MTS reagent were added into each well, and incubated for 4 h [37]. Absorbance was measured at 490/690 nm. The results are expressed as the percentage of inhibition that produced a reduction in absorbance by the CPS treatment compared to the non-treated cells. Cisplatin was used as a positive control.

#### 4.6.3. Soft Agar Assay

To estimate the effect of the CPS on colony formation (phenotype expression), the soft agar assay was performed on HL-60 cells as described [38]. Cells (2.0 × 10^4^/mL) were grown in 1 mL of 0.3% basal medium Eagle’s agar containing 10% FBS. The culture was maintained at 37 °C in a 5% CO_2_ incubator for 10 days and the cell colonies were scored using an EVOS XL Core Cell Imaging System (Thermo Fisher Scientific, Waltham, MA. USA) and the ImageJ software.

#### 4.6.4. Data Analysis

All assays were performed in three biological and two technical replicates. Results are expressed as the mean ± standard deviation (SD). Statistical differences were evaluated using the Student’s t-test and were considered significant at *p* ≤ 0.05.

## Figures and Tables

**Table 1:** ^1^H and ^13^C NMR data for the HMP, (*δ*, ppm).

Sugar Residue	H-1 C-1	H-2 C-2	H-3 C-3	H-4 C-4	H-5 C-5	H-6 C-6
→3)-α-d-GalpNAcA-(1→ **A**	5.38 99.5	4.29 49.0	3.95 77.1	4.50 70.1	4.23 72.0	174.6
→3)-β-d-GlcpNAc-(1→ **B**	4.60 103.3	3.78 55.5	3.71 81.1	3.68 72.0	3.42 76.6	3.91, 3.78 61.3

Chemical shifts of the *N*-acetyl groups are *δ_H_* 2.04–1.94 and *δ_C_* 23.4 (CH_3_), and 175.4–175.1 (CO).

**Table 2:** ^1^H and ^13^C NMR data for the OS-1 and OS-2, (*δ*, ppm).

Sugar Residue	H-1 C-1	H-2 C-2	H-3_eq,ax_ C-3	H-4 C-4	H-5 C-5	H-6_a,b_ C-6	H-7 C-7	H-8 C-8	H-9 C-9
OS-1									
→2)-β-d-Rib*p* **C**	5.13 93.8	3.77 79.3	4.17 67.8	3.83 68.6	3.87, 3.71 64.1				
→3)-β-d-Gal*p*NAcA-(1→ **D**	4.80 101.0	4.05 51.9	3.97 77.3	4.55 66.6	4.39 74.7	172.8			
→2)-α-d-Glc*p*-(1→ **E**	5.06 96.4	3.94 74.7	3.71 72.1	3.51 70.3	3.60 73.5	3.81, 3.78 61.5			
β-Pse*p*5(*R*-Lac)7Ac -(2→ **F**	173.4	101.4	2.52, 1.76 35.7	3.97 67.3	4.22 49.0	3.84 74.6	4.06 54.7	4.17 69.2	1.17 17.2
*R*-Lac-(1→	178.9	4.26 69.3	1.38 20.9						
OS-2									
→2)-β-d-Rib*p* **C**	5.13 93.8	3.78 79.3	4.18 67.8	3.83 68.7	3.87, 3.71 64.1				
→3)-β-d-Gal*p*NAcA-(1→ **D**	4.79 101.1	4.08 51.8	3.98 76.2	4.53 66.5	4.33 75.1	173.2			
→2)-α-d-Glc*p*-(1→ **E**	5.13 96.7	3.56 72.4	3.65 74.0	3.44 70.4	3.60 73.7	3.81, 3.78 61.5			

Chemical shifts of the *N*-acetyl groups are *δ_H_* 2.05–1.95 and *δ_C_* 23.6–23.3 (CH_3_), and 175.9–174.9 (CO).

**Table 3:** ^1^H and ^13^C NMR data for the MPS, (*δ*, ppm).

Sugar Residue	H-1 C-1	H-2 C-2	H-3 C-3	H-4 C-4	H-5 C-5	H-6 C-6
→3)-α-d-Gal*p*NAcA-(1→ **A**	5.19 100.4	4.26 49.6	3.96 77.0	4.56 75.7	4.19 72.1	n.d
→3)-β-d-Glc*p*NAc-(1→ **B**	n.d 104.1	n.d 55.9	n.d 81.9	3.50 72.1	3.38 77.0	3.93, 3.75 61.9
→2)-β-d-Rib*f*-(1→ **C**	5.55 105.3	4.16 84.4	4.11 72.1	3.98 83.0	3.77, 3.65 64.7	
β-d-Gal*p*NAcA-(1→ **D**	4.92 102.6	4.03 53.5	3.88 71.4	4.25 70.6	4.61 75.7	n.d

Chemical shifts of the *N*-acetyl groups are *δ_H_* 2.04–1.90 and *δ_C_* 23.5 (CH_3_), and 176.4–174.8 (CO). n.d., not determined.
